# Sex Differences in Psychostimulant Abuse: Implications for Estrogen Receptors and Histone Deacetylases

**DOI:** 10.3390/genes13050892

**Published:** 2022-05-17

**Authors:** Oscar V. Torres

**Affiliations:** Department of Behavioral Sciences, San Diego Mesa College, 7250 Mesa College Dr., San Diego, CA 92111, USA; otorres002@sdccd.edu; Tel.: +1-619-388-2292

**Keywords:** addiction, HDAC, estrogen, sex differences

## Abstract

Substance abuse is a chronic pathological disorder that negatively affects many health and neurological processes. A growing body of literature has revealed gender differences in substance use. Compared to men, women display distinct drug-use phenotypes accompanied by recovery and rehabilitation disparities. These observations have led to the notion that sex-dependent susceptibilities exist along the progression to addiction. Within this scope, neuroadaptations following psychostimulant exposure are thought to be distinct for each sex. This review summarizes clinical findings and animal research reporting sex differences in the subjective and behavioral responses to cocaine, methamphetamine, and nicotine. This discussion is followed by an examination of epigenetic and molecular alterations implicated in the addiction process. Special consideration is given to histone deacetylases and estrogen receptor-mediated gene expression.

## 1. Introduction

Over the past decades, epidemiological reports have indicated that the trajectory of substance abuse is distinct between men and women [1,2,3]. While psychological, social, and economic factors contribute to these gender disparities [4], physiological aspects also influence the onset of drug use distinctly between the sexes [5,6]. Given the conceptualization that women progress along the addiction landscape faster [7,8], it is essential to understand the physiological mechanisms that lead to sex-dependent differences in substance misuse. Thus, the focus of this review is to explore sex differences in substance abuse from a biological and intracellular perspective. Special consideration is given to clinical studies and animal research that examine gender and sex differences following cocaine, methamphetamine (METH), and nicotine exposure. Herein, *substance abuse* is defined as a chronic neuropsychiatric disorder characterized by compulsive and cyclical maladaptive behaviors of progressive drug use despite adverse consequences [9,10]. The term *gender*, referring to men or women, is used when examining results from clinical studies using human participants. The term *sex*, referring to biological features of a male or female, is used when discussing relevant findings from studies using animal models of drug addiction. The term *epigenetics* refers to regulatory processes involving posttranslational modifications (PTMs) to chromatin structure, resulting in transcription changes without altering genetic sequences [11]. Lastly, considering that psychostimulant exposure often results in adaptive PTMs such as histone acetylation and deacetylation [12], this review focuses on intracellular mechanisms associated with histone deacetylases (HDACs).

Elucidating the relation between epigenetic and intracellular mechanisms associated with substance abuse across sex is vital. First, although a large body of research examines substance abuse as a multifaceted disorder, there remains a further understanding of the molecular adaptations that occur in response to psychostimulants exposure in females. Second, because most studies from health disciplines have traditionally focused on males [13], it is essential to provide evidence that considers sex-based issues given the inequity of applying data generated from only one physiological system to both sexes. Given that female animal subjects and human participants are underrepresented, research efforts have now begun to explore sexually dimorphic variation within health complications, including addiction research, as directed by the US National Institutes of Health [14].

## 2. Neuroanatomical Correlates of Substance Abuse

Substance abuse is a neuropsychiatric condition characterized by the initiation of drug consumption, progression towards uncontrolled drug intake, and the emergence of adverse health complications [9]. Major brain structures associated with the neurobiological aspects of substance use and the subsequent development of addiction include, but are not limited to, the medial prefrontal cortex (mPFC), nucleus accumbens (NAc), and dorsal striatum [15]. The mPFC is a key structure associated with cognitive functioning such as decision making, memory retrieval, learning, and the suppression of intense emotional responses [16]. The mPFC is also implicated in the development of psychiatric disorders, including anxiety and depression [17], two major aspects of substance use. This structure receives neuronal inputs from other brain regions, including the thalamus, hippocampus, and amygdala [16]. Efferent connections, however, project excitatory inputs to the NAc [17], a neuronal pathway closely linked to the development of substance abuse. Disruption of mPFC functioning is observed in conjunction with disinhibited drug motivation and an increased drive for drug-seeking behavior [18].

The NAc is involved with motivated behaviors and the reinforcement of hedonic experiences [19]. Within this structure, drug-associated reward is manifested through dopaminergic input from the ventral tegmental area (VTA), as well as excitatory projections from the amygdala, hippocampus, thalamus, and prefrontal cortex (PFC) [20]. In addition to hippocampal activation, stimulation of the NAc following psychostimulant exposure results in an internal learning process whereby memory consolidation occurs between associated environmental cues and drug reward [21,22]. However, once learning patterns are established, there is a shift from voluntary drug usage to compulsory drug taking. This process is manifested through excitatory activation of the dorsal striatum, a brain region associated with the development of habit-forming behavior [23]. Indeed, while the dorsal striatum is linked with Parkinson’s and Huntington’s disease, it is also engaged in sequential motor-based behaviors often observed with obsessive-compulsive tendencies [24], another major indicator of drug use.

It is also noteworthy to mention the pharmacological distinctions between cocaine, METH, and nicotine which derives from the structural differences between these three drugs. In brief, cocaine enhances neuronal transmission by inhibiting the reuptake of extracellular monoamines [25]. This process results in the synaptic accumulation of dopamine, serotonin, and norepinephrine within the previously mentioned brain regions [26,27]. METH also induces the extracellular accumulation of monoamines [28]. However, this process involves vesicular monoamine transporter 2 (VMAT2) reverse transport, a mechanism resulting in redistributed dopamine from intracellular vesicular storage to excess synaptic release and neurotoxic effects [29]. In contrast, the reinforcement aspects of nicotine are manifested by activating nicotinic acetylcholine receptors (nAChRs) found along the nigrostriatal and mesolimbic dopamine pathways [30]. nAChRs are ligand-gated channels that, once stimulated by acetylcholine or exogenous agonists including nicotine, allow the influx of calcium, sodium, and potassium promoting neurotransmission via depolarized intracellular environments [31]. Thus, while these psychostimulants possess robust habit-forming and addictive properties, each has a unique mechanism of action that drives the development of substance abuse.

Within this framework, it is postulated that drug reward and hedonic experiences linked to initial substance use significantly diminish following persistent drug consumption over time [20]. Along with neurotransmitter adaptations, this descending change is thought to involve epigenetic alterations in cortical and mesolimbic systems resulting in a loss of control over drug use [32]. Together, drug-induced mPFC, NAc, and striatal dysregulation manifest adverse changes in cognitive, emotional, and motor systems, each of which ensues interconnected features of the addiction phenotype. However, evidence has accumulated indicating that these neuroadaptations may be influenced by 17β-estradiol (E_2_) [5,33,34]. This process is believed to reinforce the addictive properties of psychostimulants in a sex-dependent manner, given that, while both men and women produce E_2_, a prevalent concentration of this hormone is not observed in males but cyclically fluctuates in females.

## 3. Gender Disparities Observed in Substance Abuse

Converging lines of evidence have revealed gender differences in substance use. Traditionally, and across numerous cultures, men display higher rates of drug use in comparison to women [3]. These observations often correlate with disposable income differences, distinctions in social norms expectations, physiological variations in body weight, and drug metabolism rates across gender [4,35]. However, women may experience intensified health disturbances and adverse effects following lower drug consumption compared to men. To illustrate, women are more likely to experience secondary drug-related problems, including partner violence and sexual trauma [36,37]. In addition, women have unique drug-induced health complications such as menstrual irregularity and unfavorable pregnancy outcomes [38,39]. Relative to men, women are also more likely to report positive and pleasurable moods in response to psychostimulant exposure [40]. Women also report lower drug abstinence rates characterized by an increased likelihood of stress-induced relapse [41]. Furthermore, many psychological influences contribute to gender disparities in drug use such as early life traumas, anxiety reactivity, and adverse coping mechanisms [38,39].

Evidence also indicates that psychostimulant abuse is manifested distinctly between the sexes from a biological perspective. For example, women who use illicit psychostimulants have increased brain regions associated with addiction and decreased brain volumes associated with impulsivity control [42]. When considering the additive effect of these components, it is often conceptualized that women move forward along the course of addiction more rapidly, an observation referred to as the “telescoping effect” [1,8,43]. However, it is essential to stress that there are no certainties that predict hazardous drug use for either gender [2]. Hence, it would be inappropriate to suggest that women are simply vulnerable individuals with a greater addiction liability. Instead, understanding the specific neuro-plastic events that occur in the wake of drug exposure, between the sexes, can help develop effective therapeutic approaches for each gender.

## 4. Hormonal Influence on Substance Abuse: A Focus on Estrogen

Estrogens are essential gonadal hormones that influence growth throughout the lifespan, promote osteogenesis, mediate inflammatory responses, and impact several physiological processes including the regulation of reproductive systems [44]. The various forms of endogenous estrogen include E_2_, estrone, estriol, and estetrol [45]. As a precursor of these hormones, pregnenolone is found in the peripheral nervous system (PNS) within the gonads, adrenal glands, as well as the central nervous system (CNS), and can be derived from cholesterol through several enzymatic steps in mitochondria [46]. Following the conversion of pregnenolone to androstenedione, androstenedione then converts to either testosterone or estrone [46,47]. Testosterone can then be converted to E_2_ by aromatase and, depending on the circulating levels, estrone can also be converted to E_2_ by 17β-hydroxysteroid dehydrogenase (17β-HSD) [47]. However, in females, E_2_ is the main endogenous form of these hormones [44], with varied ovarian fluctuation across the 28-day menstrual cycle. For instance, E_2_ levels increase during the follicular phase, reach peak levels during ovulation, and decline during the luteal phase [47]. In contrast, progesterone, another ovarian hormone released in conjunction with E_2_, increases during the luteal phase [48]. E_2_ and progesterone are regulated by hypothalamic-pituitary negative feedback, while their release is stimulated via the gonadotropin-releasing, luteinizing, and follicle-stimulating hormones from the hypothalamus and pituitary glands [46,47]. In addition, E_2_ permeates the blood-brain barrier [49] and, once found in the CNS, can promote cell growth, synaptogenesis, as well as transcription [50]. Within this context, it is thought that E_2_ influences neuronal programs that impact a vast array of reward-based behaviors [51], alleviates deficits in cognition observed with neurodegenerative disease [52], and promotes memory formation [53].

Over the past 20 years, evidence has accumulated indicating that the fluctuation of ovarian hormones may be a prominent mediator of gender differences observed in substance abuse [6,33,54]. Specifically, E_2_ is noted to enhance reactions to rewarding stimuli [51] and amplify the hedonic effects of psychostimulants [5,34]. For instance, elevated levels of E_2_ correlate with enhanced positive subjective moods following psychostimulant exposure in women [54,55]. Animal studies examining sex differences in cocaine, METH, or nicotine have also revealed that E_2_ has an essential role in mediating the rewarding aspects of these drugs. In female rodents, fluctuations in ovarian hormones occur across proestrus, estrus, metestrus, and diestrus, four distinct phases of the estrous cycle [56]. E_2_ levels are highest during proestrus, decline during estrus, and remain relatively low during metestrus and diestrus [57]. In agreement with clinical reports, increased E_2_ levels also correlate with enhanced drug-seeking behaviors in female rodents [33]. Thus, the following sections provide selective summaries of clinical studies and findings from animal research comparing the subjective and behavioral effects of cocaine, METH, and nicotine between the sexes.

### 4.1. Gender Differences in Cocaine Use

Cocaine is an illicit psychostimulant with a robust addictive profile [25]. Cocaine addiction is characterized by recurrent patterns of drug use and negative health consequences [58]. Regarding gender differences, women progress faster through the stages of cocaine addiction and exhibit shorter cocaine-abstinence periods than men [59]. While environmental stressors such as the occurrence of early traumatic events [60] contribute to gender disparities in cocaine use, hormonal distinctions between men and women also play an important role. For instance, the hedonic impact of cocaine is increased in a menstrual phase-dependent manner, as women seeking cocaine report an enhanced “high” during the follicular phase of the menstrual cycle [41]. These findings are further corroborated by evidence demonstrating that smoked cocaine induces elevated positive subjective effects in women during the follicular phase of the menstrual cycle [61,62]. Conversely, the administration of progesterone, which reduces the effects of E_2_, is reported to decrease the positive subjective effects of cocaine in women, an effect not observed in men [63,64]. However, there is evidence indicating no differences in the subjective effects of cocaine across the menstrual cycle [65]. Interestingly, the route of cocaine administration in human participants (e.g., intranasal vs. intracranial) has been noted as a potential confounding variable when considering the relation between E_2_ and enhanced cocaine reward [65].

### 4.2. Sex Differences in the Behavioral Response to Cocaine

Multiple research laboratories have compared the reinforcing effects of cocaine across sex using the intravenous self-administration (IVSA) paradigm. The IVSA paradigm is a preclinical assessment used to examine the motivational and reinforcing properties of drugs in animals. This paradigm utilizes operant conditioning whereby responses, as noted by “active” lever presses or nose pokes, result in light/tone cues and programed drug delivery via intravenous infusions. Inactive responses, as noted by presses on a non-drug associated lever, result in no consequences. The paradigm can consist of several phases including acquisition, escalation, maintenance, extinction, and reinstatement of drug seeking by cue- or drug-induced methods [66,67]. Many researchers have noted that female rodents display enhanced addiction profiles relative to male rodents, using IVSA procedures. For example, female rats acquire cocaine IVSA faster [68] and respond more under progressive ratio (PR) schedules of reinforcement than male rats [69]. Female rats also respond more for cocaine under extended access [70,71] or short-access IVSA paradigms compared to male rats [72]. Under a PR schedule, female rats display higher breaking point averages for cocaine than males [73,74]. Enhanced addiction profiles are also observed in female rodents during adolescence. For instance, adolescent female rats display faster acquisition for cocaine IVSA under fixed ratio (FR) and PR schedules, compared to male adolescents [75]. The notion that females may experience greater cocaine reward is also observed across species. Specifically, cocaine self-administration (SA) is enhanced in female cynomolgus monkeys compared to male cynomolgus monkeys [76], an effect attributed to fluctuations in ovarian hormones during the follicular phase. Female rats also respond more to cocaine-associated cues following a withdrawal period (incubation of cocaine craving) than male rats [69,77]. These effects are thought to be mediated via hormonal changes throughout the estrous cycle. For instance, following either a 15- or 48-day absence period from cocaine IVSA, female rats in estrus display enhanced cue-induced drug-seeking behavior [77,78]. Even after the extinction of cocaine IVSA has occurred, female rats demonstrate greater cocaine-induced reinstatement during estrus [79,80].

The role of E_2_ in mediating cocaine reward is further illustrated by studies utilizing ovariectomized (OVX) procedures in female rodents. For example, OVX female rats display reductions in cocaine IVSA compared to free-cycling female rats [81]. Furthermore, estrogen benzoate treatment recuperates cocaine IVSA in OVX females [81]. E_2_ treatment in OVX female rats also results in faster acquisition of cocaine IVSA compared to control OVX rats [70,82,83]. In contrast, progesterone is thought to counteract the effects of E_2_ on cocaine IVSA. For instance, OVX female rats simultaneously treated with E_2_ and progesterone display lower escalation for cocaine IVSA than OVX female rats treated with E_2_ only [82,84]. E_2_ also seems to have a unique effect on brain-associated neurochemical responses to cocaine. Specifically, OVX female rats treated with E_2_ benzoate display cocaine-induced dopamine increases within the dorsal striatum, an effect not observed in castrated male rats [85]. Castrated male rats treated with E_2_ also display an increased preference for cocaine than vehicle-treated castrated male rats [86]. Moreover, by using four core genotypes (FCG) mice, a mouse model designed to generate XY mice exhibiting ovaries and XX mice exhibiting testes [87], it was demonstrated that male XY mice with ovaries acquire cocaine IVSA faster than gonadal-intact males [88]. Together, multiple studies have demonstrated sex differences in cocaine IVSA. These effects are likely mediated by E_2_-associated processes leading to enhanced drug-seeking behaviors.

### 4.3. Gender Differences in Methamphetamine Use

METH is another illicit psychostimulant with a strong potential for abuse liability [89]. Addiction to METH is characterized by a rapid escalation of drug intake, loss of control over drug usage, and the emergence of cognitive deficits [90]. Regarding gender differences, women tend to use METH at an earlier age [91] and become addicted to METH at a faster rate [92]. Additionally, women are more likely to experience comorbid psychiatric symptoms in conjunction with METH use [93]. Psychological and social factors associated with early METH use among women include weight reduction motivations, intimate partner influences, and physical abuse [93,94,95]. METH use distinctly impacts men and women from a physiological perspective as well. For instance, amongst METH users, women tend to experience greater METH dependence [92], develop thinner frontal cortices, have larger NAc volumes, and display greater impulsivity compared to men [41]. However, men tend to have greater amphetamine-induced striatal dopamine release compared to women [96]. Gender disparities in METH abuse might be associated with the effects of hormonal fluctuations across the menstrual cycle. For example, women report an increase in positive mood and euphoria following amphetamine exposure during the follicular phase of the menstrual cycle [97,98]. In healthy women, E_2_ treatment also increases the positive subjective experiences of amphetamine [99]. However, there is evidence indicating that progesterone enhances the positive effects of amphetamine in non-addicted women [100]. Collectively, these reports demonstrate that varying levels of gonadal hormones in women alter the subjective rewarding effects of METH.

### 4.4. Sex Differences in the Behavioral Response to Methamphetamine

Preclinical studies investigating sex differences in METH IVSA have revealed inconsistent findings. For example, evidence indicates that female rats acquire METH SA significantly faster than male rats under FR1 conditions and 6-h access to the drug [101]. Under similar conditions, female rats display faster escalation for drug intake and self-administer more METH than male rats [102]. Compared to male rats, female rats also demonstrate enhanced motivation for METH SA, as noted by an increase in responses for the drug under FR5 conditions [103,104]. In contrast, there is evidence indicating that male rats intake more METH than female rats under 6-h extended access procedures using FR1 regimens [105,106]. However, other reports have noted no sex differences in METH SA when using short [107,108] or extended access [109,110,111] IVSA procedures. Interestingly, under a 96-h METH IVSA access paradigm, female rats display escalation patterns characterized by a three-fold increase in their METH intake, an effect not observed for male rats [112]. After extinction procedures, female rats also respond more for METH following cue-induced or drug-primed reinstatement procedures [107,113,114]. Additionally, male and female rats demonstrate similar incubation of METH seeking following a 30-day abstinence period from METH SA [105,106,111]. Along with distinct METH IVSA procedures, drug dose regimens might account for some of these inconsistencies, given that female rats display greater responses for lower METH doses [101] than male rats [105,106].

E_2_ is also thought to mediate some of the sex-specific behavioral responses to METH. For instance, METH SA is lower in OVX female rats in comparison to E_2_-treated OVX female rats [115]. Utilizing the conditioned place preference (CPP) paradigm, another animal model of drug abuse, it is reported that E_2_ treatment in OVX female mice facilitates METH-induced CPP in comparison to castrated male mice [116]. Taken together, these studies report that females display greater METH seeking than male rodents, and implicate E_2_ as a potential mediator of METH addiction-like behavior in a sex-dependent manner.

### 4.5. Gender Differences in Nicotine Use

Although the prevalence of smoking cigarettes has declined within the US over the past 50 years [117], tobacco remains a commonly used drug coupled with powerful addiction properties. As a health concern, smoking tobacco is linked to many medical complications, including cardiovascular failure, lung disease, and is associated with the development of cancer [118]. While there is a multitude of chemical components found within a given cigarette, nicotine is generally considered one of the primary habit-forming substances linked to the rewarding aspects of smoking [119,120]. Regarding sex differences, clinical studies have observed gender disparities in tobacco use. For example, men tend to consume more cigarettes than women [121]. However, women are less likely to benefit from smoking cessation aids [122], have lower success rates when attempting to quit smoking [123], and report more nicotine withdrawal symptoms during smoking abstinence [124]. Clinical reports indicate that some of the gender disparities in tobacco use may be, in part, linked to ovarian hormone fluctuations across the menstrual cycle. For instance, earlier reports suggested that cigarette use increases during menses [125], and the craving for smoking increases during the luteal phase [125]. However, more recent reports indicate that enhanced nicotine use among women increases when E_2_ levels are highest [126,127]. In contrast, the average number of cigarettes smoked per day decreases when progesterone levels are increased [128]. In addition, women seeking treatment for nicotine dependence display an increased likelihood of smoking abstinence when progesterone levels are highest [129]. Thus, fluctuations in ovarian hormones affect cigarette use, craving for smoking, and increase the propensity for relapse [127,130].

### 4.6. Sex Differences in the Behavioral Response to Nicotine

Animal research has also observed sex differences in nicotine SA. For example, the acquisition of nicotine IVSA is faster in female rats relative to male rats [131,132]. Multiple studies utilizing IVSA procedures have also demonstrated that female rats intake more nicotine than male rats [133,134,135]. Under an FR5 schedule, female rats also display more nicotine IVSA compared to males [136]. Nonetheless, there are reports indicating that male rats acquire nicotine IVSA faster than females [72], and no sex differences in nicotine IVSA [137]. Data from animal studies also demonstrate the importance of ovarian hormones in mediating nicotine reward. For instance, E_2_ differentially enhances nicotine-induced striatal dopamine release between the sexes, with female rats displaying greater dopamine release than males [138]. Additionally, female rats respond more to nicotine SA under PR schedules during estrus [132]. Compared to regular cycling female rats, OVX females display reductions in nicotine-induced place preference [139] and nicotine IVSA [133,140]. These effects are estrogen-related, given that E_2_ treatment partially recovers nicotine SA in OVX female rats [133,140]. However, variations across the estrous cycle may not influence nicotine reward in female rats when considering findings from the nicotine IVSA [131] or CPP paradigms [139].

## 5. Histone Acetylation as a Regulatory Mechanism of Gene Expression

Gene expression is regulated through dynamic PTMs to the chromatin structure that restricts or provides access to DNA. Transcriptional activation and silencing are processes modulated, in part, by the epigenetic modification of histones via reversible interactions between their N-terminal tails and nuclear enzymes [141,142]. Within chromatin, each nucleosome consists of an individual octameric structure made of four main histone proteins, including H2A, H2B, H3, and H4 (with 2 of each primary histone), wrapped around 147 base pairs of DNA [142,143]. Each histone protein contains lysine residues that undergo PTMs. Negatively charged DNA structures form an electrostatic force with positively charged lysine residues, forming a compressed state [141,144]. Large protein complexes containing enzymes known as histone acetyltransferases (HATs) can add acetyl groups to this structure, leading to a neutralized electrostatic state resulting in chromatin relaxation [144,145]. This process reduces DNA affinity toward nucleosomes, thus rendering promoter regions more accessible [144,145]. By contrast, repressor complexes containing enzymes known as histone deacetylases (HDACs) remove acetyl groups [146,147]. The removal of acetyl groups results in N-terminal tail electrostatic charge and chromatin compression. This process restricts transcription factors from accessing promoter regions along DNA sequences, leading to gene silencing [148]. However, while these dynamic processes are associated with transcriptional stimulation and repression, it is important to note that in some cases histone deacetylation can prompt transcriptional activation [147].

## 6. Histone Deacetylases and Drug Exposure

Currently, there are 18 known mammalian HDACs categorized into four distinct family classes. These include the zinc-dependent Class I (HDAC 1, 2, 3, 8), Class IIa (HDAC 4, 5, 7, 9), Class IIb (HDAC 6 and 10), and the Class IV (HDAC 11) family member [149]. The Class III HDACs are nicotinamide adenine dinucleotide (NAD)1-dependent enzymes called Sirtuins and have seven distinct subfamily members [146]. Relevant to this review, HDACs are broadly expressed throughout the brain and serve vital roles including intracellular regulation, cell differentiation, cell viability, and are implicated in drug-induced gene expression. Specifically, it is thought that HDACs function to regulate the transcriptional responses necessary for the formation of molecular and intracellular environments conducive to the addiction phenotype following drug exposure [150,151].

### 6.1. Class I Histone Deacetylases

Identified during the mid-1990s, HDAC1 and HDAC2 are nuclear-localized enzymes with an estimated 80% shared sequence homology [152,153]. These enzymes are essential for embryonic development, with their deletion resulting in early lethality [154]. In addition, both enzymes contain a single deacetylase domain, share similar intracellular functions including DNA repair [152,153,154], and engage in protein-protein associations with large transcriptional repressive complexes, including Swi-independent 3 (Sin3) [155,156], repressor element-1 silencing transcription corepressor (CoREST) [154], and nucleosome remodeling deacetylase (NuRD) [157]. Together, these protein complexes regulate gene silencing and activation via the remodeling of chromatin accessibility [157]. HDAC1 and HDAC2 also mediate behavioral responses to psychostimulant exposure. For instance, the non-specific HDAC inhibitors (HDACi) phenylbutyrate and trichostatin A (TSA) have been found to attenuate cocaine SA in rats [158]. Furthermore, conditional knockdown of HDAC1 within the NAc reduces cocaine-induced locomotor sensitization in mice [151]. In relation to METH exposure, an acute METH challenge results in overexpression of *Hdac1* and *Hdac2* in the mouse mPFC [159]. However, acute METH exposure decreases HDAC1 and increases HDAC2 expression in the rat NAc [160]. Furthermore, conditional knockdown of HDAC2 prolongs the overexpression of immediate early genes in the mouse NAc following acute METH exposure [161]. In relation to nicotine exposure, treatment with the HDACi sodium butyrate facilitates the extinction of nicotine SA in rats [162]. In addition, nicotine-induced CPP is associated with increased HDAC2 expression in the rat NAc [163].

HDAC3, identified shortly after HDAC1 and HDAC2 [164,165], is another nuclear Class I family member that is abundantly expressed in the brain [166] and shares structural similarities to HDAC8 [149]. HDAC3 is involved in embryonic development, DNA repair, and long-term memory formation [167]. Similar to HDAC1 and HDAC2, HDAC3 induces transcriptionally repressive activity however it associations with other protein complexes including nuclear receptor corepressor (NCoR) and silencing mediator of retinoid and thyroid hormone receptors (SMRT) [168]. Furthermore, HDAC3 coimmunoprecipitates with HDAC4 and HDAC5 such that the enzymatic activity of these other family members is dependent on the presence of HDAC3 [169]. In relation to psychostimulant exposure, systemic HDAC3 inhibition results in the suppression of cocaine SA reinstatement [170]. However, the latter report also notes that HDAC3 inhibition does not alter cocaine SA under PR or FR schedules of reinforcement. Selective inhibition of HDAC3 also facilitates the extinction of cocaine-induced CPP in mice [171]. Furthermore, local deletion of HDAC3 in the NAc facilitates cocaine-induced CPP in mice [172]. Chronic cocaine exposure alters the deacetylase activity of HDAC3 and the transcriptional expression of HDAC-target genes in the mouse NAc in a cell-specific manner [173]. However, local knockdown of HDAC3 in the NAc does not reduce cocaine locomotor sensitization [151]. With respect to METH exposure, a single METH challenge decreases *Hdac3* mRNA in the NAc [174]. However, repeated METH exposure does not alter *Hdac3* mRNA in the mPFC [159]. In the dorsal striatum, Fos-positive neurons overexpress *Hdac3* mRNA following a 30-day withdrawal period from METH SA in rats [175]. Regarding nicotine exposure, phenylbutyrate treatment, another HDACi, results in reduced nicotine-induced CPP in rats [163].

HDAC8, originally identified by multiple researchers in the year 2000 [176,177], is another important Class I family member known for its role in cellular proliferation and oncogene control [178]. Aside from being found in the cell nucleus, HDAC8 can translocate to the cytoplasm and deacetylate non-histone targets, including tumor protein P53 (p53), α-actin, and cAMP response element-binding protein (CREB) [179]. This activity occurs in the absence of large complexes, given that, unlike other Class I family members, HDAC8 lacks a C-terminal tail, used to recruit corepressors [180]. Because of its non-histone targets, HDAC8 is implicated in the regulation of signaling pathways, such as mitogen-activated protein kinase (MAPK) and activator protein 1 (AP-1) [178]. Interestingly, TSA or phenylbutyrate treatment reduced cocaine SA and reinstatement in rats [158,181]. Regarding METH exposure, non-contingent chronic METH challenges decrease *Hdac8* mRNA levels in the dorsal striatum of mice [182]. Similarly, a single METH challenge decreases *Hdac8* mRNA levels in the mouse NAc [174]. However, a single METH challenge increases *Hdac8* mRNA levels in the mouse mPFC [159], suggesting distinct regional responses to METH. To date, the potential effects of HDAC8 on nicotine action are not fully understood.

### 6.2. Class II Histone Deacetylases

Class IIa HDACs have received substantial focus considering their shuttling ability between nuclear and cytoplasmic compartments [183]. HDAC4, HDAC5, and HDAC6 possess a C-terminal catalytic domain similar to Class I HDACs, but also have an additional N-terminal domain that is able to bind onto transcription factors, leading to gene silencing [184]. Upon dephosphorylation, Class IIa HDAC family members enter the cell nucleus and become enzymatically active by combining with NCoR and SMRT complexes containing HDAC3 [169]. However, once their N-terminal serine residues become phosphorylated, Class IIa HDAC family members transport out of the nucleus and return to the cytoplasmic compartment via 14-3-3 adaptor proteins [184]. This translocation prevents access to core histones, which results in hyperacetylation and the expression of genes. Interestingly, HDAC4, HDAC5, and HDAC9 become degraded within the cell nucleus, while HDAC7 undergoes cytoplasmic degradation following phosphorylated-induced nuclear export [185].

HDAC4, first identified in 1999 [186], is a potent transcriptional repressor with high expression within skeletal muscle, heart, and neuronal tissues [166]. Within the brain, HDAC4 is involved in the development of spatial learning and memory processes [186], and plays important biological roles including apoptosis, cell differentiation, and neurogenesis [187,188]. Moreover, the nuclear and cytoplasmic concentration of HDAC4 is varied within distinct cell types of the CNS, with phosphorylation leading to cytoplasmic localization [189]. Differentiated cleaved isoforms of HDAC4 also determine the intracellular localization of this enzyme [184]. HDAC4 is known to associate with HDAC3 and the SMRT/NCoR complex [169]; however, the enzymatic properties of HDAC4 are inactive in the absence of HDAC3 [190]. Concerning drug exposure, non-contingent cocaine administration leads to hyperphosphorylation of HDAC4 and subsequent nuclear export of this enzyme in striatal mouse tissue [191]. Pharmacological degradation of HDAC4 is associated with enhanced cocaine SA [192], while viral overexpression of HDAC4 in the NAc is associated with reduced cocaine SA [193]. Additionally, knockout (KO) of HDAC4 within the NAc of mice results in a reduction of cocaine-induced CPP and locomotor responses [191]. Regarding METH, a single METH challenge results in decreased *Hdac4* mRNA level in the NAc of mice [174]. However, single and repeated METH challenges increase *Hdac4* mRNA levels in the mPFC of mice [159].

HDAC5, identified along with HDAC4 and HDAC6 [186], is another important Class IIa HDAC family member implicated in transcriptional repression, microtubule regulation, and axonal growth [194]. The intranuclear shuttling of HDAC5 involves protein kinase C subtype μ (PKCμ), calcium/calmodulin-dependent protein kinase II (CaMK-II), and protein kinase A (PKA) [195]. For instance, HDAC5 phosphorylation by PKCμ leads to 14-3-3 chaperone interactions and cytoplasmic localization [196]. Moreover, PKA can prevent this process by phosphorylating HDAC5 resulting in nuclear import [197]. Regarding psychostimulant exposure, chronic cocaine exposure increases HDAC5 phosphorylation and cytoplasmic localization in the mouse NAc [198], a process implicating CaMK-II. In addition, viral overexpression of nuclear-bound HDAC5 within the NAc reduces cocaine-induced CPP in mice [197]. Within the striatum, cocaine exposure leads to the nuclear import of dephosphorylated HDAC5 via protein phosphatase 2 (PP2A) [199]. Following cocaine sensitization, *Hdac5* mRNA expression is decreased within the dorsal striatum and PFC [200]. Regarding METH exposure, viral-induced overexpression of HDAC5 in the rat dorsal striatum results in enhanced METH seeking after prolonged drug abstinence following IVSA procedures [201]. Interestingly, the same study reports that dorsal striatal knockdown of HDAC5 results in decreased METH seeking after a drug abstinence period [201]. With respect to nicotine, increases in global levels of H3 and H4 acetylation are observed in the mouse striatum following nicotine exposure, a result thought to be mediated by the inhibitory effects of nicotine on HDAC activity [202].

HDAC7 plays a vital role in cardiovascular viability, immune cell response [203], and apoptotic neuronal protection [169]. Relative to the expression levels of other HDAC family members in the CNS, HDAC7 has the lowest expression [166]. This Class IIa family member has independent deacetylase action [204], but also possesses deacetylation properties within nuclear and cytoplasmic compartments when associated with HDAC3 [169]. Intracellular signaling and nuclear shuttling of HDAC7 involves protein kinase D (PKD), whereby phosphorylation of HDAC7 induces nuclear export, and CaMK-I promotes cytoplasmic localization [183]. In addition, HDAC7 binds with NCoR and SMRT [169] and has both transcriptional activation and repressive properties. In relation to psychostimulant exposure, treating human primary astrocytes with cocaine results in upregulated HDAC7 protein levels [205]. Local knockdown of HDAC2 in the NAc results in upregulation of *Hdac7* in mice [151]. Similarly, HDAC2 KO mice demonstrate increased *Hdac7* mRNA levels following an acute METH challenge [161].

HDAC9, cloned in early 2001 [206], is another important Class IIa family member involved in the regulation of T-cell immune responses [207], metabolism of lipids, development of atherosclerosis plaques, and vascular inflammation [208]. Within the CNS, HDAC9 is observed in mature neurons, but not in supporting glial cells including astrocytes or oligodendrocytes [209]. In regard to psychostimulant exposure, HDAC9 KO mice do not display differences in cocaine-induced CPP relative to control mice [198]. The latter study also reports that overexpression of HDAC9 in the mouse NAc has no effect on cocaine reward. Related to METH exposure, a single METH challenge does not alter *Hdac9* mRNA levels in the mouse NAc [161].

Like other Class II family members, HDAC6 imports to the cell nucleus, where it interacts with distinct large protein complexes such as ligand-dependent corepressor (LCoR) [210]. However, HDAC6 is predominantly found within the cytoplasmic compartment, where it interacts with non-histone substrates, including heat shock protein (Hsp)90, extracellular-signal-regulated kinase (ERK) 1, and α-tubulin [211]. The distinct biological roles of HDAC6 include microtubule stabilization, cell division, cell migration, and cytoskeletal dynamics, including the elimination of misfolded proteins due, in part, to its zinc finger-ubiquitin binding protein (ZnF-UBP) domain [212]. Because of its prominent role in the regulation of aggresomal formation, HDAC6 has been implicated as a potential therapeutic target for various neurodegenerative disorders characterized by a cognitive decline [213]. Regarding psychostimulant exposure, HDAC6 levels are increased in the rat PFC following synthetic cannabinoid and cocaine exposure, an effect not observed when challenging rats with cocaine only [214]. With respect to METH, systemic pharmacological inhibition of HDAC6 results in decreased METH IVSA in rats [215]. In relation to nicotine, exposing lung cancer cells to nicotine results in the inhibition of HDAC6 activity [216].

HDAC10, identified in 2002 [217,218], shares structural homology with HDAC6 and possesses two distinct catalytic domains. Specifically, HDAC10 contains an N-terminal catalytic domain and a secondary C-terminal leucine-rich catalytic domain [217]. HDAC10 serves distinct biological functions, including DNA repair and mitotic regulation [219]. In addition to nuclear localization, HDAC10 can be found in the cytosolic compartment, where its C-terminal leucine-rich domain allows for cytoplasmic accumulation [218]. However, once imported to the cell nucleus, HDAC10 interacts with SMRT, HDAC3, and HDAC2 to commence transcriptional repression [169]. Regarding METH exposure, a single METH challenge does not alter *Hdac10* mRNA levels in the mouse mPFC [159]. Similarly, a single METH challenge does not alter *Hdac10* mRNA expression within the mouse NAc [174]. However, repeated METH challenges reduce *Hdac10* mRNA levels within the rat dorsal striatum [182]. With respect to nicotine, exposing human neuroblastoma cells to nicotine results in nucleosome repositioning and *Hdac10* DNA accessibility [220].

### 6.3. Class IV Histone Deacetylases

HDAC11, identified in 2002, is the only current member of the Class IV family and is the most abundantly expressed HDAC in the rat brain [166,221]. HDAC11 is the smallest of the HDACs [221] and interacts with corepressor complexes containing HDAC6 and HDAC9 [222]. This enzyme has an important role in regulating inflammatory T cell response, maintaining metabolic homeostasis [223] and fatty acid deacetylase activity [224]. Relative to other HDAC family members, the enzymatic preference of HDAC11 for histone lysine residues is thought to be relatively lower [225]. In addition, HDAC11 has been found in both nuclear and cytoplasmic compartments; however, this localization may be cell type-dependent [226]. Regarding drug exposure, HDAC11 expression has been found to increase following cocaine SA in the rat dorsal striatum, cingulate cortex, and NAc [226]. In relation to METH exposure, *Hdac11* mRNA levels decrease in the rat dorsal striatum following a 30-day withdrawal period from METH IVSA [227]. *Hdac11* mRNA levels also decrease in the rat dorsal striatum following repeated METH challenges [182]. In contrast, *Hdac11* mRNA decreases in the mouse NAc following acute METH exposure [174].

## 7. Intracellular Mechanisms of Estrogen Receptors

**Figure 1:**
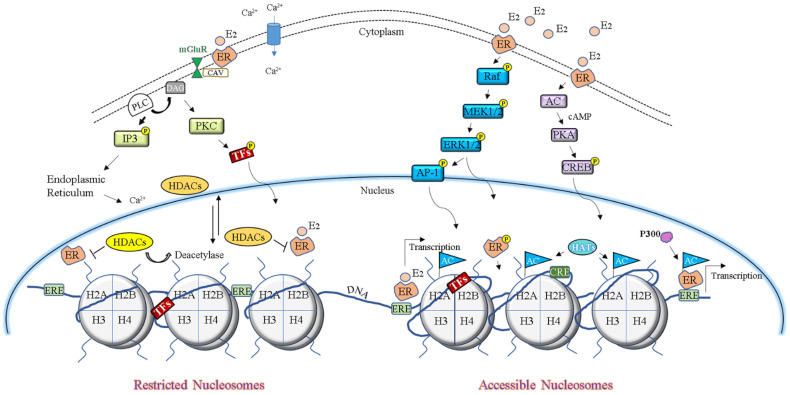
Conveys the epigenetic mechanisms discussed in this review. Intracellular signaling cascades following estrogen receptor and 17β-estradiol (ER/E_2_) associations and the effects of histone deacetylases (HDACs) on chromatin are displayed. On top, the schematic displays cell membrane-bound ER activated by E_2_ and possible downstream intracellular pathways leading to gene expression. Illustrated on the top-left, metabotropic glutamate receptor (mGluR) and ER-mediated activation is indicated via caveolin protein interactions as well as intracellular calcium influx. Additionally, E_2_ activation of the phospholipase C (PLC) cascade is demonstrated. On the top-right portion of the schematic, E_2_ activation of the protein kinase A (PKA) and mitogen-activated protein kinase/extracellular-signal-regulated kinase (MAPK/ERK) cascades are depicted. Here, cell membrane-bound ERs indirectly influence nuclear environments by the phosphorylation and activation of cAMP response element-binding protein (CREB), leading to CRE-mediated gene expression. Similarly, stimulation of the MAPK/ERK cascades and subsequent phosphorylated activator protein 1 (AP-1) is displayed as another intranuclear regulation by membrane-bound ERs. The bottom of the schematic depicts distinct molecular environments surrounding nucleosomes that generally limit or promote gene expression. Illustrated on the bottom-left are nucleosomes with densely packed DNA, characterized by repressed access to promoter regions and estrogen response element (ERE) sites. Here, nuclear, and cytoplasmic-shuttling, HDACs perform an integral role in restricting transcription by removing histone acetyl groups. HDACs are also depicted as blocking ERs from reaching their genomic targets. On the bottom-right portion of the schematic, nucleosomes are depicted with relaxed states, characterized by loosely wrapped DNA. Here, histone acetyltransferases (HATs) add acetyl groups to lysine residues, resulting in reduced DNA-histone affinity and accessible ERE sites. Classical ER-mediated transcription is illustrated via E_2_ activated nuclear ER targeting an ERE site. A phosphorylated ER is also depicted as an additional mechanism of transcriptional activation. Furthermore, the acetylation of ERs by p300 is depicted as another possible mechanism that promotes transcription within the accessible nucleosome state. Collectively, these epigenetic mechanisms and molecular adaptations may drive enhanced drug-seeking behavior more so in females compared to males.

The molecular activity of E_2_ is mediated by the estrogen receptor (ER) α and the ER β, two distinct steroid hormone receptors [228,229]. Here, (ERs) is used when referencing both ERα and ERβ subtypes. Both ERs share amino acid sequence similarities [228] and are cell membrane-localized or nuclear-bound [229]. ERα was identified during the 1960s [230,231] and cloned during the late 1980s [232]. ERβ was characterized and cloned in the mid-1990s [233,234]. In addition, ERβ has five distinct isoforms [235], while ERα has two distinct isoforms [236]. In the brain, ERs are found within neurons and glial cells [47,236] and serve diverse biological functions including cell survival, neuronal plasticity, gene regulation, and memory formation [53]. ERs possess six distinct domains denoted as A/B to F [237]. The A/B domains contain activation function (AF)-1 and mediate transcription in a ligand-dependent and -independent manner. In the absence of E_2_, ERs can become activated via phosphorylation by kinases such as ERK and phosphatidylinositol 3-kinase (PI3K) [238]. The C domain consists of two different zinc-fingers that allow ERs to recognize and bind onto DNA [239]. Furthermore, ERs have a high affinity for estrogen response elements (EREs), a 17-bp palindromic DNA sequence (5′-GGTCA—TGACC-3′) [237,240] found along various genes [228]. Once bound to either full ERE sites or partial ERE half-sites [241], these receptors recruit RNA polymerase II and assemble large activator complexes to stimulate transcription [237]. While both receptors share overlapping ERE targets, they also independently prefer specific gene promoters and display competitive ERE selection, as each can displace the other from ERE sites, resulting in reduced transcriptional activity [241]. In this manner, ERα and ERβ play essential roles in regulating global patterns of gene expression, via direct nuclear mechanisms and transcriptional programs. Domains D/E include AF-2 [242] and regulate transcription via non-classical mechanisms by interacting with either transcriptional co-activator or co-repressive proteins. While AF-1 functions without ligand binding, AF-2 is ligand-dependent [228]. Ligand-linked ERs can form homodimers or heterodimers that recruit transcriptional machinery to AP-1 responsive elements [229] or activate diverse protein-kinase cascades, including PKA, MAPK/ERK, and phospholipase C (PLC) [243], which induce CREB phosphorylation resulting in gene expression [244]. Furthermore, cell membrane-localized ERs also induce the rapid release of intracellular Ca+ stores [245], triggering multiple G-protein-related intracellular signaling cascades and pathways. ERs also modulate transcriptional activity in a ligand-independent manner when their lysine residue becomes acetylated or their serine residues become phosphorated [244]. Moreover, through membrane caveolin protein interactions, ER can couple with metabotropic glutamate receptors (mGluRs), resulting in CREB phosphorylation (see Figure 1 for an illustrated representation depicting some of the reviewed molecular mechanisms by which E_2_ stimulates transcriptional responses) [243,246].

However, ligand-linked ERs can also suppress transcription by targeting genes lacking an ERE site through associations with HDAC-containing complexes [238] such as LCoR, SMRT, and metastasis-associated protein 1 (MTA-1) [247]. Additionally, ligand-linked ERβ is known to inhibit transcription through AP-1 sites [248]. Thus, ERs indirectly suppress or enhance gene expression through alternative promoter regions [228]. The F domain contains a C-terminal able to enhance overall DNA-binding capacity [249]. This aspect is of interest given that lysine residues along ER domains can be reversibly acetylated or deacetylated, a process involving HAT and HDAC activity. To illustrate, p300 acetylates K266/K268 and K302/K303 found along the DNA-binding domain of ERα, whereas TSA exposure halts the deacetylation of ERα [249,250,251]. However, each acetylated lysine pattern impacts transcriptional activity distinctly, as acetylated K266/288 enhances ERα-mediated gene expression while K302/303 acetylation decreases transcription [249,251].

## 8. Estrogen Receptors and Sex Difference in Neural Adaptations following Drug Exposure

Studies using animals have noted that ERβ plays a prominent role in mediating addiction-like behavior linked to cocaine exposure. For example, following extinction from cocaine IVSA, systemic administration of diarylpropionitrile, an ERβ agonist, enhances cocaine seeking during reinstatement procedures in OVX female rats [84]. Interestingly, this same effect was not observed when administering an ERα agonist [84]. Similarly, local infusions of diarylpropionitrile into the prefrontal cortex potentiates drug-seeking behavior in OVX female rats following extinction from cocaine IVSA [252], an effect not observed when locally administering an ERα agonist into the same region. Female mice treated with an ERβ agonist also display increases in cocaine-induced CPP, while ERβ knockdown in the NAc decreases cocaine-induced CPP [253]. Furthermore, local administrations of a G protein-coupled estradiol receptor (GPER) agonist into the dorsolateral striatum enhances breaking point averages and potentiates reinstatement of cocaine IVSA in female but not male rats [74]. These effects are most likely associated with E_2_-mediated enhancement of cocaine reward through ER activation, given that E_2_ treatment enhances cocaine SA in OVX female rats and not in castrated male rats [82].

Relative to cocaine, fewer reports have examined the neuro-plastic and molecular adaptations following METH exposure between sex. For instance, female rats exhibit increases in synaptic activity within the prelimbic area of the PFC, as noted by evoked excitatory currents following METH IVSA [110]. Following METH IVSA, and incubation of METH seeking, female rats display increased corticotropin-releasing hormone receptor 2 mRNA levels in the hippocampus and decreased prodynorphin mRNA levels in the PFC [105]. However, male rats display increased prodynorphin mRNA levels in the NAc after similar METH SA procedures, an effect not observed in female rats [106]. Male rats also display hippocampal brain derived neurotrophic factor (BDNF) increases following METH IVSA procedures, an effect not observed in female rats [109]. Collectively, these studies demonstrate sex-dependent changes in response to METH exposure across distinct brain regions. Given that varying levels of E_2_ distinctly affect dopaminergic and glutamatergic transmission in reward circuitry [254], ER/E_2_ interactions can influence intracellular mechanisms associated with METH addiction distinctly between males and females. This is probable, as E_2_ administration has a greater effect on striatal dopamine release following amphetamine exposure in OVX females compared to castrated male rats [255]. In addition, E_2_ can influence reward-linked behaviors in a sex-dependent manner [245] through cell membrane ER-metabotropic glutamate receptor associations [256]. Although non-genomic, stimulating metabotropic glutamate receptors by ligand-bound ERs induces MAPK and CREB phosphorylation [257], which in turn stimulate transcriptional responses. Furthermore, E_2_ stimulated CREB phosphorylation may be sex-specific, as only hippocampal neurons from female rats exhibit this effect [243]. These observations are relevant given that proteins along the MAPK/ERK intracellular cascade are phosphorated following METH IVSA [258]. E_2_ might also exert neuroprotective elements that are sex-dependent. For instance, E_2_ treatment prior to challenging mice with neurotoxic METH doses reduces the amount of dopamine depletion in the striatum of OVX female mice, an effect not observed for male mice [259].

The ability of ER/E_2_ interactions to enhance nicotine reward also includes dopaminergic and glutamatergic transmission along mesolimbic systems. Within the NAc, multiple dendritic spines found on medium spiny neurons (MSNs) receive dopamine and glutamate inputs [260]. Dopamine input originates from the VTA, and glutamatergic inputs derive from the PFC, hippocampus, and amygdala [261]. The necks of dendritic spines receive input from dopamine projections, while spine heads receive glutamatergic input [262]. Following nicotine exposure, nAChRs activate and stimulate excitatory projections to the NAc, including those of dopaminergic producing neurons from the VTA [263]. This process promotes drug-induced neuro-plastic events in MSNs such as dendritic growth and synaptic formation. However, females may experience an increased sensitivity to estradiol-induced plasticity, given that women display higher synaptic spine densities within the NAc than men [264]. Additionally, women display a higher concentration of E_2_ in the NAc, along with a wider distribution of E_2_ in multiple neurons compared to men [264]. Moreover, in female rats, E_2_ stimulates greater nicotine-induced dopamine release within the striatum, an effect not observed in male rats [138]. Interestingly, nicotine and its metabolite cotinine can prevent aromatase activity causing the inhibition of E_2_ syntheses, a process known to occur within the brain [265]. In addition, nicotine can inhibit ERE binding capacity within the CNS [266]. Thus, the effect of E_2_-ER interactions on nicotine reward most likely involves membrane-bound ER and disrupted associations in intracellular cascades. The observation that OVX female rats demonstrate diminished nicotine IVSA [133] is also of interest, given that OVX procedures cause the downregulation of ERβ in the NAc and VTA [140]. Similar to the partial recovery of nicotine SA in OVX rats following E_2_ treatment [133], decreased NAc ERβ levels are also recuperated in OVX rats following E_2_ treatment [140].

## 9. Estrogen Receptor and HDAC Interactions

Given the limited literature on the effects of psychostimulant exposure and cellular adaptations involving E_2_ and HDAC interactions, the following section provides a brief discussion covering HDACs-ER associations within the scope of cancer research. Multiple HDAC family members are implicated as therapeutic targets for the treatment of tumor growth [267]. This research branch has led to an accumulation of data that characterizes the interplay between ERs and HDACs, given their regulatory roles over gene expression [268]. For example, in human breast cancer cells, ERα recruits HDAC1 and reduces p53-mediated transcription [269]. In addition, ERα recruits NCoR and SMRT, resulting in transcriptional silencing, a process indicating ER-HDAC3 associations [269]. In agreement with the latter observation, ERα links with HDAC3 and forms a repressive HDAC3/ERα complex [270]. These findings indicate that ERs can recruit HDACs and stimulate their silencing activity. However, not all ER-HDAC interactions result in transcriptional repression. For example, HDAC6 can bind onto the AF-2 domain of ERα and form an HDAC6/ER complex that deacetylates tubulin [271]. Several HDAC family members also regulate the activity of ERs. For instance, HDAC1 interacts with the AF-2 domain and reduces ER-mediated transcription in breast cancer cells [272]. Additionally, HDAC4 suppresses the transcriptional activity of nuclear ERα in an E_2_-dependent and independent manner [273]. Similarly, HDAC7 and HDAC9 can lower the expression and transcriptional activity of ERα within breast cancer cells [274,275]. These findings indicate that, although ERs recruit the epigenetic silencing actions of HDACs, HDACs can modulate the pro-transcriptional properties of ERs.

This notion is further sustained by studies using HDACi to examine the effects of deacetylated ERs in cancer cells. For example, TSA treatment enhances ER-mediated transcriptional activity in breast cancer cells [276]. Treating breast cancer cells with TSA also induces the nuclear translocation of ERβ, suggesting an increase in transcriptional functionality [276]. In corroboration with the latter observation, treating glioblastomas with panobinostat or romidepsin (two HDACi) upregulates the expression and promotes the activation of ERβ [277]. Furthermore, exposing T5 human cancer cells to E_2_ results in the rapid acetylation of core histones, a process associated with reduced HDAC activity [278]. However, treating these cells with tamoxifen, an anti-estrogen cancer medication, results in transcriptional silencing via the recruitment of multiple HDAC-containing complexes [279]. Thus, in the absence of HDAC activation, it is likely that E_2_ facilitates cellular growth through histone accessibility and ER-induced transcription.

Related to the notion, E_2_ promotes synaptogenesis and enhances pro-neuro plastic processes associated with memory formation [52,280]. For instance, within the hippocampus, E_2_-ER interactions increase synaptic protein immunoreactivity, enhance dendritic spine density, and stimulate synaptic button outgrowth [281]. Because E_2_ levels fluctuate in females, these processes may represent an intracellular environment conducive to sex-dependent transient neuronal plasticity and structural expansion. Indeed, while men typically have larger gray matter volumes within the hippocampus, women undergo hippocampal volume increases during the late follicular phase [282], a period of relatively high levels of E_2_. In agreement with the latter observation, converging lines of data from neuroimaging studies report that ovarian E_2_ release drives structural neuro-plastic expansion within the hippocampus, hypothalamus, NAc, and amygdala [48], all brain regions associated with the addiction process. Evidence from animal research further demonstrates that dendritic spine densities on MSNs are enhanced within the NAc of female compared to male rodents [283]. Female rats also have more dopaminergic neurons in the VTA and display greater dopamine transmission in the striatum, including dopaminergic release, receptor density, and binding capacity, compared to males [254]. Interestingly, varying levels of E_2_ influence membrane-bound ER expression. Specifically, the expression levels of both ERs increase during estrus and lower during proestrus [284].

Considering the evidence described above, a plausible mechanism may exist whereby ER-driven synaptic stimulation ensues the strengthening of memory consolidation for drug-associated cues in a sex-dependent manner. This process most likely involves periods of histone accessibility through PTMs and disrupted HDAC activity following drug exposure. In this context, unabated ER-mediation transcription and cyclical E_2_ fluctuations might further promote a neuro-plastic environment that potentiates drug reward in females. Based on the molecular mechanisms reviewed herein, Figure 1 illustrates cytoplasmic and nuclear E_2_-ER interactions that induce transcriptional activity. As previously discussed, E_2_ is associated with the enhancement of hedonic stimuli [51] and facilitates the rewarding aspects of psychostimulants [128]. However, additional investigations are necessary to fully clarify the associations between ERs, HDAC activity, and their influence on substance abuse vulnerability.

## 10. Concluding Remarks and Future Considerations

In conclusion, clinical studies have identified differences in substance abuse between men and women. Animal research has provided data indicating that, amongst other physiological factors, E_2_ has a significant role in mediating these sex differences. In addition, HDACs contribute to the development of addiction-like behaviors through the reversible accessibility and restriction of histones. This process includes epigenetic regulation of transcriptional codes following drug exposure. Based on the evidence examined in this review, it is speculated that sex differences in substance abuse might involve HDAC-ER interactions, which alter gene expression along neurological pathways associated with addiction. E_2_ fluctuations may further cyclically influence hazardous drug use for females within this framework. Moreover, Class II HDACs represent prominent targets to examine, given their dynamic responses following psychostimulant exposure and regulation over ER-mediated transcription. Altogether, this review supports the notion that substance abuse is a chronic neuropsychiatric disorder characterized by intracellular responses that may be distinct for each sex. The value of integrating female-specific findings in addiction research is also highlighted, given that sex-based comparisons in epigenetic adaptations remain relatively unexplored. This issue is important, as a better understanding of the mechanisms that mediate sex differences in substance abuse is needed to develop gender-appropriate treatment practices for patients seeking rehabilitation and recovery options.

## Abbreviations

17β-HSD	17β-hydroxysteroid dehydrogenase
AF	activation function
AP-1	activator protein 1
BDNF	brain derived neurotrophic factor
CaMK	calcium/calmodulin-dependent protein kinase
CNS	central nervous system
CoREST	REST (repressor element-1 silencing transcription) corepressor
CPP	conditioned place preference
CREB	cAMP response element-binding protein
E_2_	17β-estradiol
ER	estrogen receptor
EREs	estrogen response elements
ERK	extracellular-signal-regulated kinase
FCG	four core genotypes
FR	fixed ratio
GPER	G protein-coupled estradiol receptor
HATs	histone acetyltransferases
HDAC	histone deacetylase
HDACi	HDAC inhibitor
Hsp90	heat shock protein
IVSA	intravenous self-administration
KO	knockout
LCoR	ligand-dependent corepressor
MAPK	mitogen-activated protein kinase
METH	methamphetamine
mGluRs	metabotropic glutamate receptors
mPFC	medial prefrontal cortex
MSNs	medium spiny neurons
MTA-1	metastasis-associated protein 1
NAc	nucleus accumbens
nAChRs	nicotinic acetylcholine receptors
NAD	nicotinamide adenine dinucleotide
NCoR	nuclear receptor corepressor
NuRD	nucleosome remodeling deacetylase
OVX	ovariectomized
p53	tumor protein P53
PFC	prefrontal cortex
PI3K	phosphatidylinositol 3-kinase
PKA	protein kinase A
PKCμ	protein kinase C subtype μ
PKD	protein kinase D
PLC	phospholipase C
PNS	peripheral nervous system
PP2A	protein phosphatase 2
PR	progressive ratio
PTMs	posttranslational modifications
SA	self-administration
Sin3	Swi-independent 3
SMRT	silencing mediator of retinoid and thyroid hormone receptors
TSA	trichostatin A
VMAT2	vesicular monoamine transporter 2
VTA	ventral tegmental area
ZnF-UBP	zinc finger-ubiquitin binding protein
